# Impact of gender as a prognostic factor in HBV-related Hepatocellular Carcinoma: the survival strength of female patients in BCLC stage 0-B

**DOI:** 10.7150/jca.33430

**Published:** 2019-07-10

**Authors:** Lihua Yu, Xiaoli Liu, Xinhui Wang, Zhibo Dang, Yuyong Jiang, Xianbo Wang, Zhiyun Yang

**Affiliations:** 1 Center of Integrative Medicine, Beijing Ditan Hospital, Capital Medical University, Beijing 100015, P.R. China; 2 First Clinical Medical College, Beijing University of Chinese Medicine, Chaoyang District, Beijing 100029, P.R. China

**Keywords:** HBV-related Hepatocellular carcinoma, Barcelona Clinic Liver Cancer classification, Gender, Risk factors

## Abstract

**Background and Aims**: Although previous studies suggested that female patients who underwent curative resection in early-stage hepatocellular carcinoma (HCC) had better survival rates than male patients, it is unclear whether females in different HCC stages actually have survival advantage. This study aimed to investigate whether gender differences in the Barcelona Clinic Liver Cancer (BCLC) classification system contributed to different survival outcomes in hepatitis B virus (HBV)-related HCC.

**Methods**: A retrospective analysis was performed of 1,753 patients diagnosed with HBV-related HCC between January 2008 and June 2017 at the Beijing Ditan hospital. The BCLC stages were classified into BCLC stage 0-B and BCLC stage C-D groups. Factors determining overall survival (OS) and progression-free survival (PFS) were analyzed via univariate and multivariate analysis using the Kaplan-Meier method and Cox proportional-hazards regression models.

**Results**: The cohort consisted of 1,202 BCLC stage 0-B and 551 BCLC stage C-D HBV-related HCC patients. Gender was identified to be an independent risk factor for OS (HR = 0.617; 95% CI, 0.426-0.895; *p* = 0.011) and PFS (HR = 0.728; 95% CI, 0.558-0.950; *p* = 0.019) in BCLC stage 0-B HBV-related HCC patients. With respect to OS and PFS, there were significant differences between female and male patients only in BCLC stage 0-B, but not in BCLC stage C-D. The OS and PFS in BCLC stage 0-B for female patients was significantly greater than that for male patients (*p* = 0.0103, *p* = 0.0112). Tumor multiplicity and size were independent risk factors for female patients in BCLC stage 0-B, whereas tumor multiplicity, tumor size, HBV-DNA, hemoglobin, total bilirubin, and alpha-fetoprotein levels were independent risk factors for male patients in BCLC stage 0-B.

**Conclusions**: Different outcomes in OS or PFS with respect to gender only exist in BCLC stage 0-B HBV-related HCC patients. Female patients have a better outcome than male patients in BCLC stage 0-B.

## Introduction

Hepatocellular carcinoma (HCC) is the second-most common cause of cancer death worldwide. Based on the 2012 global statistics, China alone accounted for about 50% of the total number of HCC cases and deaths. Hepatitis B virus (HBV) infection is the main cause of HCC 1-4. Since at first most patients were vaguely diagnosed with intermediate or advanced HCC, the Barcelona Clinic Liver Cancer (BCLC) staging system provided a better prediction value for patients with disease diagnosis at a very early stage 5-6. Therefore, the BCLC staging system is now widely used in the diagnosis and evaluation of HCC.

Gender differences persist in the incidence of HCC, with males being the predominant population. Different male to female ratios can also be observed in different locations 4. Sex hormones play an important role in these gender-related incidences i.e., androgens stimulate the androgen signaling pathway while estrogen probably plays a protective role during HCC development or outcome 7-9. However, there has been some controversy about the role of gender with respect to HCC prognosis in recent years. Several studies showed that female patients had better survival rates than male patients after surgical resection 10-12. Contrastingly, a recent study consisting of different etiologies and ethnicities showed that female patients were not an independent predictor for survival in HCC patients 14. Therefore, the prognostic effect of gender in HCC remains inconsistent. Unfortunately, only a few studies about the impact of gender on HBV-related HCC have been carried out.

The purpose of our study is to find the impact of gender on the survival and progression of different BCLC-staged HBV-related HCC.

## Materials and Methods

### Study population

We performed a retrospective cohort study on 1,753 HBV-related HCC patients using data from the Beijing Ditan Hospital between January 2008 and June 2017. Patients with HBV-HCC were defined as those who had serum that was positive for the hepatitis B surface antigen (HBsAg; ≥ 6 months) and conformed to the HCC diagnosis. We reviewed the data according to the 2017 China guidelines to confirm HCC diagnosis 15. The HCC diagnosis data included biopsy, radiology, and alpha-fetoprotein (AFP) serology. First, we selected patients based on hepatic angiography, pathology, or AFP ≥ 400 μg/L in combination with ultrasonography, computed tomography (CT), and magnetic resonance imaging (MRI). Next, we only included patients with complete clinical data and more than a year of follow-up. Our exclusion criteria included: (1) other viral infections such as human immunodeficiency virus (HIV) or hepatitis C virus (HCV); (2) hepatitis caused by non-phagocytic hepatitis; (3) metastatic liver cancer; (4) pregnant women; (5) incomplete data; (6) patients with less than a year of follow-up; and (7) patients with unclear BCLC staging.

### Definition of BCLC stages

The Barcelona Clinic Liver Cancer (BCLC) staging system includes variables related to tumor stage, liver functional status, physical status, and cancer-related symptoms (2011 AASLD) 16. Very early stage (BCLC 0) is single HCC (< 2 cm) and currently very difficult to diagnose accurately. Early stage (BCLC A) is solitary HCC or up to 3 nodules (≤ 3 cm) and exhibits liver function (Child-Pugh A and B). The intermediate stage (BCLC B) consists of large/multifocal liver cancer patients with no tumor-related symptoms, no macrovascular invasion, or extrahepatic diffusion and includes Child-Pugh A and B patients. The advanced stage (BCLC C) comprises of patients who present with cancer symptoms and/or vascular invasion or extrahepatic spreading. The end stage (BCLC D) consists of patients with extensive tumor involvement leading to severe physical condition (PS score > 2) and/or severe damage to liver function (Child-Pugh C). In this study, we compared the demographic data and clinical factors across 2 groups (BCLC stage 0-B and BCLC stage C-D).

### Demographic and clinical data

We extracted the following data for the study: gender, age, family history of HBV, alcohol use, smoking history, HBV-DNA, hepatitis B e-antigen (HBeAg) at baseline, Child-Pugh score, tumor multiplicity, tumor size, portal vein tumor thrombus (PVTT) at baseline, cirrhosis, white blood cell (WBC), red blood cell (RBC), hemoglobin (HGB), platelet (PLT), creatinine (CR), alanine aminotransferase (ALT), total bilirubin (TBIL), albumin (ALB), γ-glutamyl transferase (r-GGT), prothrombin (PTA), and alpha-fetoprotein (AFP) levels. Age at the time of HCC diagnosis. Hepatitis B status was determined by the presence of HBsAg or HBV-DNA, or via documented history of HBV infection. Laboratory values at the time of HBV-related HCC diagnosis were classified as elevated when they were more than the clinical normal values. The BCLC staging system was used to determine the tumor stage, and Child-Pugh scores were used to determine liver function.

### Statistical analysis

Demographic data and clinical factors were compared across the 2 groups (BCLC 0-B and BCLC C-D) using the χ^2^ test or Fisher's exact probability test for categorical variables. Overall survival (OS) and progression-free survival (PFS) rates were calculated using the Kaplan-Meier method. Differences between the curves were assessed according to the log-rank test. OS was calculated from the date of diagnosis to the date of death regardless of reason of death or date of last follow-up. PFS was calculated from the date of diagnosis to the date when tumor progress was diagnosed or date of last follow-up.

Cox proportional regression was used to identify the gender factors that were independently associated with BCLC stage 0-B HBV-related HCC. In order to assess the gender-independent prognostic factors, variables that were identified to be significant in the univariate analysis were included in the multivariate Cox proportional-hazards model. Differences with *p* < 0.05 were considered to be statistically significant.

We used Kaplan-Meier curves to estimate OS and PFS between BCLC stage 0-B and BCLC stage C-D HBV-related HCC patients, and compared the survival curves of the 2 groups using the log-rank test. Differences in survival among female and male patients within different BCLC stages were also evaluated using the Kaplan-Meier method and log-rank test. All statistical analyses were performed using SPSS 22.0.

## Results

### Baseline characteristics of the BCLC stage 0-B and BCLC stage C-D groups

Table 1 displays the demographic and clinical characteristics of the 2 groups based on BCLC stage. The cohort consisted of 1,202 BCLC stage 0-B and 551 BCLC stage C-D HBV-related HCC patients. No significant differences between the BCLC stage 0-B and BCLC stage C-D were observed with regard to gender at the time of induction (male, 78.4% vs 78.8%; *p* = 0.851), patients older than 50 years of age (72.0% vs 70.4%, *p* = 0.506), and family history of HBV (68.1% vs 68.1%, *p* = 0.974). However, a significantly greater proportion of HBV-HCC patients in BCLC stage 0-B were non-smokers, non-alcoholic, HBV-DNA (≤ 500 IU/mL), Child-Pugh A, tumor solitary, tumor size (< 5 cm), without PVTT at baseline, and without cirrhosis when compared to the BCLC stage C-D group. The baseline laboratory values for HGB, PLT, and PTA were higher in the BCLC stage 0-B patients when compared to the BCLC stage C-D patients. RBC, CR, ALT, TBIL, ALB, r-GGT, and AFP levels were lower in BCLC stage 0-B patients when compared to the BCLC stage C-D patients.

### Multivariate analyses of prognostic factors for OS and PFS in BCLC stage 0-B and BCLC stage C-D groups

The independent prognostic factors related to OS and PFS in BCLC stage 0-B in the multivariate analysis are listed in Table 2. Multivariate analysis indicated that factors such as female (HR = 0.617; 95% CI, 0.426-0.895; *p* = 0.011), HBV-DNA (> 500 IU/mL; HR = 1.472; 95% CI, 1.121-1.933; *p* = 0.005), tumor multiplicity (HR = 1.550; 95% CI, 1.188-2.024; *p* = 0.001), tumor size (> 5 cm; HR = 2.636; 95% CI, 1.995-3.484; *p* < 0.0001), HGB level (> 120 g/L; HR = 0.638; 95% CI, 0.483-0.843; *p* = 0.002), and AFP level (> 400 ng/mL; HR = 2.235; 95% CI, 1.696-2.947; *p* < 0.0001) were independent risk factors for OS; whereas female (HR = 0.728; 95% CI, 0.558-0.950; *p* = 0.019), HBV-DNA (> 500 IU/mL; HR = 1.537; 95% CI, 1.274-1.855; *p* < 0.0001), tumor multiplicity (HR = 1.369; 95% CI, 1.132-1.657; *p* = 0.001), smoking (HR = 1.289; 95% CI, 1.054-1.577; *p* = 0.014), HGB level (> 120 g/L; HR = 0.609; 95% CI, 0.498-0.743; *p* < 0.0001), and AFP level (> 400 ng/mL; HR = 1.971; 95% CI, 1.605-2.420; *p* < 0.0001) were independent risk factors for PFS.

The independent prognostic factors related to OS and PFS in BCLC stage C-D in the multivariate analysis are listed in Table 3. Multivariate analysis indicated that factors such as HBV-DNA (> 500 IU/mL; HR = 1.284; 95% CI, 1.001-1.646; *p* = 0.049), PVTT at baseline (HR = 1.490; 95% CI, 1.135-1.956; *p* = 0.004), HGB level (> 120 g/L; HR = 0.702; 95% CI, 0.548-0.899; *p* = 0.005), PLT level (> 100 × 10^9^/L; HR = 1.739; 95% CI, 1.358-2.227; *p* < 0.0001), TBIL level (> 18.8 μmol/L; HR = 1.397; 95% CI, 1.058-1.845; *p* = 0.018), and AFP level (> 400 ng/mL; HR = 1.591; 95% CI, 1.236-2.048; *p* < 0.0001) were independent risk factors for OS; whereas PVTT at baseline (HR = 1.370; 95% CI, 1.104-1.699; *p* = 0.004), PLT level (> 100 × 10^9^/L; HR = 1.452; 95% CI, 1.190-1.772; *p* < 0.0001), ALT level (> 50 U/L; HR = 1.317; 95% CI, 1.083-1.601; *p* = 0.006), and AFP level (> 400 ng/mL; HR = 2.141; 95% CI, 1.743-2.629; *p* < 0.0001) were independent risk factors for PFS.

**Fig 1:**
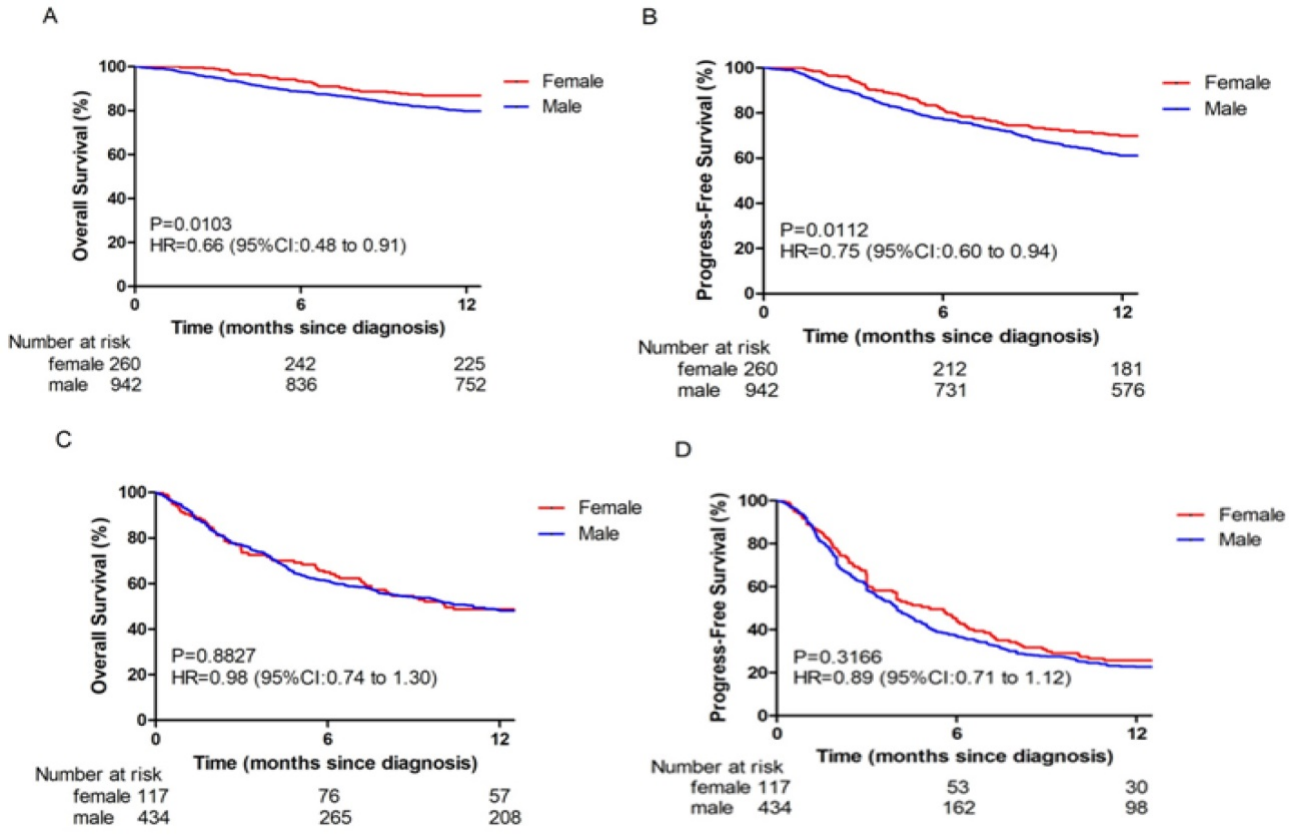
Overall survival (OS) and Progression-free survival (PFS) in different BCLC stage HBV-related HCC patients. (A-B) The OS (A) and PFS (B) in BCLC stage 0-B. (C-D) The OS (C) and PFS (D) in BCLC stage C-D.

In this study, gender was an independent predictor for survival in BCLC stage 0-B. The 1-year OS (86.5%) and PFS (70.0%) rates for female patients were better than those of male patients (79.8% and 61.1%, respectively; *p* = 0.0103 and *p* = 0.0112 respectively; Fig 1A and 1B). However, no significant differences between female and male OS (48.7% vs 47.9%, respectively; *p* = 0.8827; Fig 1C) and PFS (25.6% vs 22.6%, respectively; *p* = 0.3166; Fig 1D) rates were observed in BCLC stage C-D.

Meanwhile, in our subsequent prospective cohort study, there are 264 patients in BCLC stage 0-B HBV-related HCC, including 213 male and 51 female. Supplementary Table 2 displays the results of multivariable analyses. Female was independent prognostic factors for OS (HR = 0.336; 95% CI, 0.131-0.859; *p* = 0.023) and PFS (HR = 0.536; 95% CI, 0.292-0.985; *p* = 0.044) in the BCLC stage 0-B HBV-related HCC patient. The 1-year OS (90.2%) and PFS (76.5%) rates for female patients were better than those of male patients (75.6% and 58.2%, respectively; *p* = 0.031 and *p* = 0.026, respectively; Fig S1A and S1B).

### Multivariate analyses of predictive factors for female and male patients in BCLC stage 0-B

Supplementary Table 1 presents the BCLC stage 0-B patient characteristics with respect to gender groups. BCLC stage 0-B HBV-HCC patients include 260 female and 942 male patients with a female:male ratio of 1:3.6. Compared to the male group, the female group consisted of more patients older than 50 years of age (*p* < 0.0001), non-smokers (*p* < 0.0001), non-alcoholics (*p* < 0.0001), solitary tumors (*p* = 0.034), and smaller tumors (*p* = 0.035). Furthermore, female patients had lower laboratory values for HGB (*p* < 0.0001), PLT (*p* < 0.0001), ALT (*p* = 0.001), and r-GGT (*p* < 0.0001) when compared to male patients.

Multivariate analysis showed that tumor multiplicity (*p* = 0.036) and tumor size (*p* = 0.001) were independent risk factors for OS in female patients, whereas HBV-DNA level (*p* = 0.016), tumor multiplicity (*p* = 0.008), tumor size (*p* < 0.0001), HGB level (*p* = 0.001), TBIL level (*p* = 0.023), and AFP level (*p* < 0.0001) were independent risk factors for OS in male patients (Table 4).

**Fig 2:**
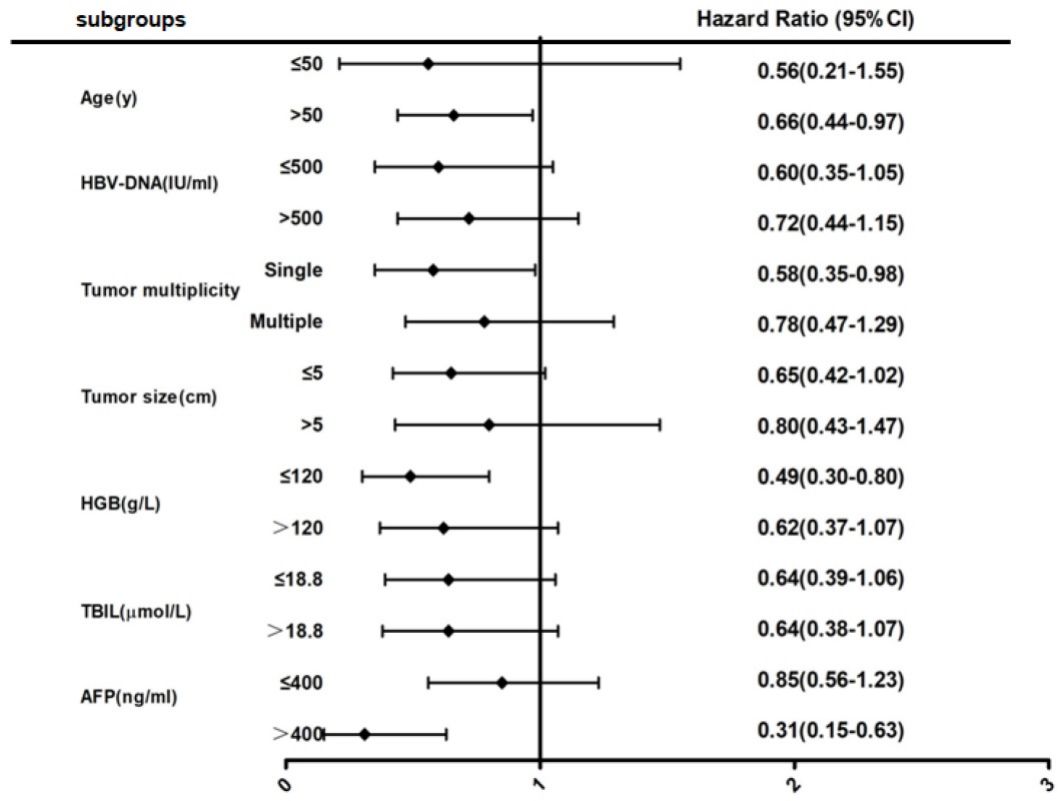
Forest map comparing mortality risk between female and male group in BCLC stage 0-B HBV-related HCC patients.

After multivariable analyses (Fig 2), subgroup analysis showed that female patients over 50 years of age had a 34% lower risk of death when compared to male patients (HR = 0.66; 95% CI, 0.44-0.97). Female patients with solitary tumors also had a 42% lower risk of death when compared to male patients (HR = 0.58; 95% CI, 0.35-0.98). Furthermore, female patients with low hemoglobin had a 51% lower risk of death or high AFP levels had a 69% lower risk of death when compared to male patients (HR = 0.49; 95% CI, 0.30-0.80 or HR = 0.31; 95% CI, 0.15-0.63, respectively).

## Discussion

Previous studies have shown that gender disparity is not only associated to HCC incidence, but is also reflective of HCC prognosis. Female patients have been shown to have lower incidences of HCC and better survival rates than male patients 10-12. Further studies revealed that female patients at early stages (TNM system stage I/II) had better survival rates than male patients after curative HCC treatments 11-13. Another study also found that female HCC patients had less invasive symptoms when compared to male HCC patients 12, which was consistent with our findings. When survival was analyzed according to the BCLC staging system, our results showed that female patients in the BCLC stage 0-B HCC stage had better survival rates than the male patients in the same stage. Furthermore, female patients in BCLC stage 0-B had a lower risk of death with increased age (> 50 years), solitary tumors, high AFP levels (> 400 ng/mL), and low HGB levels (< 120 g/L) when compared to male patients.

An observation of the HCC diagnosis age showed that female patients were older than male patients, likely because estrogen played a protective role against HCC development 14, 17. Naugler et al. reported that the gender disparity in HCC was due to the differences in MyD88-dependent IL-6 production 18. Meanwhile, Prieto J proposed that the estrogen-mediated inhibition of IL-6 production by Kupffer cells (KCs) reduced HCC risk in females, and that these findings could be used to prevent HCC in males 19. Furthermore, Montella M et al. and Li, C et al. found that androgens promoted the increased transcription and replication of HBV genes in HBV-related HCC patients by stimulating its signaling pathway, and that estrogen could play a protective role against the progression of HBV infections by decreasing HBV RNA transcription and inflammatory cytokine production 20-21. In our study, young female patients (< 50 years) showed lower HCC incidence rates, which could be due to the protective effect of estrogen. However, older female patients (> 50 years) showed a lower risk of death when compared to male patients in BCLC stage 0-B HBV-related HCC, probably because the protective effect of estrogen is still existent. Therefore, estrogen not only reduces the incidence of liver cancer, but also provides better prognosis for HBV-HCC. However, none of the above results were observed in younger female patients as the number of younger female patients were significantly lesser than the older ones.

Alpha-fetoprotein (AFP) levels are considered to be indicators of tumor activity in HCC patients 23. A previous study reported that serum AFP levels had considerable predictive value with respect to the malignant features and prognosis of HCC 22. However, AFP levels have also been reported to have no prognostic value in well-compensated cirrhotic patients with single, small HCC treated with curative intent 24. Moreover, AFP levels were found to not correlate with the tumor load in female patients 12, 24. Although the ability of AFP levels to predict HCC prognosis may be controversial, AFP has been shown to be affected by the condition of the tumor, including the tumor size and tumor multiplicity 25. In this study, AFP served as a prognostic factor only in the surviving male BCLC stage 0-B HBV-HCC patients. Although AFP levels were higher in the female patients than in the male patients, most of the female patients had normal liver function, tumor sizes < 5 cm, and solitary tumors. Interestingly, these findings were consistent with a previously reported study 12.

Anemia is a common hematological abnormality in patients with cancer, which leads to cancer progression and reduced survival 26. The presence of anemia increased the risk of death by 65% and shortened the survival time of patients with cancer when compared to those who did not have anemia 27-28. However, in our study, female HBV-related HCC patients with anemia had a significantly lower risk of death when compared to male HBV-related HCC patients with anemia. Female patients were also found to have lower hemoglobin levels than male patients and the results were consistent with a previous study 14. Tanaka et al. and Bachman E et al. reported that testosterone stimulated erythropoietin (EPO) production and increased iron utilization to support the increase in hemoglobin levels 29-30. We suspect that it may be this physiological characteristic in males that causes the above results. Hemoglobin is a protective factor, and female patients may able to adapt to lower hemoglobin levels when compared to male patients.

Several limitations exist in our retrospective study. Firstly, since only patients with HBV-associated HCC were included, the study was lacking in the other causes of HCC, such as hepatitis C virus, alcoholic liver, and non-alcoholic fatty liver. Secondly, since younger female patient (< 50 years) numbers were insufficient, notable differences with respect to age were not observed. Because the protective effect of estrogen is obvious, future studies should include female HCC patients from younger demographics. Finally, our study primarily focused on the short-term survival outcome. Longer observation periods may possibly give rise to differences in outcome with respect to gender.

In conclusion, we found that gender affected the short-term survival outcome in BCLC stage 0-B HBV-related HCC patients. Therefore, BCLC stage 0-B HBV-HCC patients, especially males who are at high risk of HCC, need to undergo regular screening.

## Supplementary Material

Supplementary figure and tables.

## Figures and Tables

**Table 1:** Demographic data and clinical characteristics of patients with HBV-related hepatocellular carcinoma

Demographic and clinical values		BCLC 0-B n=1202 (%)		BCLC C-D n=551 (%)		p Value
Gender						0.851
Male		942 (78.4)		434 (78.8)		
Female		260 (21.6)		117 (21.2)		
Age(y)						0.506
≤50		337 (28.0)		163 (29.6)		
>50		865 (72.0)		388 (70.4)		
Smoking						0.039
Non-smoker		741 (61.6)		311 (56.4)		
Smoker		461 (38.4)		240 (43.6)		
Alcohol						<0.0001
No alcohol		796 (66.2)		317 (57.5)		
Alcohol		406 (33.8)		234 (42.5)		
Family history of HBV						0.974
Yes		819 (68.1)		375 (68.1)		
No		383 (31.9)		176 (31.9)		
HBV-DNA(IU/ml)						<0.0001
Low≤500		673 (56.0)		196 (35.6)		
High>500		529 (44.0)		355 (64.4)		
HBeAg at baseline						0.609
Negative		837 (69.6)		377 (68.4)		
Positive		365 (30.4)		174 (31.6)		
Child Staging						<0.0001
A		777 (64.6)		126 (22.9)		
B		425 (35.4)		206 (37.4)		
C		0 (0.0)		219 (39.7)		
Tumor multiplicity						<0.0001
Solitary		765 (63.6)		243 (44.1)		
Multiple		437 (36.4)		308 (55.9)		
Tumor size(cm)						<0.0001
≤5		987 (82.1)		328 (59.5)		
>5		215 (17.9)		223 (40.5)		
PVTT at baseline						<0.0001
Yes		9 (0.7)		353 (64.1)		
No		1193 (99.3)		198 (35.9)		
Cirrhosis						<0.0001
Yes		1095 (91.1)		534 (96.9)		
No		107 (8.9)		17 (3.1)		
WBC (109/L)						0.311
Low≤4		502 (41.8)		216 (39.2)		
High>4		700 (58.2)		335 (60.8)		
RBC (109/L)						<0.0001
Low≤4		504 (41.9)		368 (66.8)		
High>4		698 (58.1)		183 (33.2)		
HGB(g/L)						<0.0001
Low≤120		348 (29.0)		294 (53.4)		
High>120		854 (71.0)		257 (46.6)		
PLT (109/L)						0.002
Low≤100		611 (50.8)		323 (58.6)		
High>100		591 (49.2)		228 (41.4)		
CR(μmol/L)						<0.0001
Normal≤111		1172 (97.5)		514 (93.3)		
High>111		30 (2.5)		37 (6.7)		
ALT (U/L)						<0.0001
Normal ≤50		892 (74.2)		343 (62.3)		
High>50		310 (25.8)		208 (37.7)		
TBIL(μmol/L)						<0.0001
Normal≤18.8		743 (61.8)		138 (25.0)		
High>18.8		459 (38.2)		413 (75.0)		
ALB(g/L)						<0.0001
Normal≤40		786 (65.4)		494 (89.7)		
High>40		416 (34.6)		57 (10.3)		
r-GGT(U/L)						<0.0001
Normal≤60		704 (58.6)		172 (31.2)		
High>60		498 (41.4)		379 (68.8)		
PTA(%)						<0.0001
Low≤70		305 (25.4)		311 (56.4)		
High>70		897 (74.6)		240 (43.6)		
AFP(ng/ml)						<0.0001
Low≤400		962 (80.0)		322 (58.4)		
High>400		240 (20.0)		229 (41.6)		

p Value between BCLC 0-B and BCLC C-D groups. PVTT, portal vein tumor thrombus; WBC, white blood cell; RBC, Red blood cell; HGB, haemoglobin; PLT, platelet; CR, creatinine; AFP, alpha-fetoprotein; ALT, alanine aminotransferase; TBIL, totall bilirubin; ALB, albumin; γ-GGT, γ-glutamyl transferase; PTA, prothrombin time activity.

**Table 2:** Multivariate analysis of risk factors related to overall survival and progression-free survival in BCLC stage 0-B HBV-related HCC patients

Variable	Overall Survival				Progression-free Survival		
	HR	95%CI	P		HR	95%CI	P
Gender							
Male	1				1		
Female	0.617	0.426-0.895	0.011		0.728	0.558-0.950	0.019
Smoking							
Non-smoker	----				1		
Smoker	----	----	----		1.289	1.054-1.577	0.014
HBV-DNA(IU/ml)							
Low≤500	1				1		
High>500	1.472	1.121-1.933	0.005		1.537	1.274-1.855	<0.0001
Tumor multiplicity							
Solitary	1				1		
Multiple	1.55	1.188-2.024	0.001		1.369	1.132-1.657	0.001
Tumor size(cm)							
≤5	1				----		
>5	2.636	1.995-3.484	<0.0001		----	----	----
HGB(g/L)							
Low≤120	1				1		
High>120	0.638	0.483-0.843	0.002		0.609	0.498-0.743	<0.0001
AFP(ng/ml)							
Low≤400	1				1		
High>400	2.235	1.696-2.947	<0.0001		1.971	1.605-2.420	<0.0001

HGB, haemoglobin; AFP, alpha-fetoprotein; Cox proportional hazards model; P < 0.05 was considered as statistically significant.

**Table 3:** Multivariate analysis of risk factors related to overall survival and progression-free survival in BCLC stage C-D HBV related HCC patients

Variable	Overall Survival				Progression-free Survival		
	HR	95%CI	P		HR	95%CI	P
HBV-DNA( IU/ml)							
Low≤500	1				----		
High>500	1.284	1.001-1.646	0.049		----	----	----
PVTT at baseline							
Yes	1				1		
No	1.49	1.135-1.956	0.004		1.37	1.104-1.699	0.004
HGB(g/L)							
Low≤120	1				----		
High>120	0.702	0.548-0.899	0.005		----	----	----
PLT (10^9^/L)							
Low≤100	1				1		
High>100	1.739	1.358-2.227	<0.0001		1.452	1.190-1.772	<0.0001
TBIL(μmol/L)							
Normal≤18.8	1				----		
High>18.8	1.397	1.058-1.845	0.018		----	----	----
ALT (U/L)							
Normal ≤50	----				1		
High>50	----	----	----		1.317	1.083-1.601	0.006
AFP(ng/ml)							
Low≤400	1				1		
High>400	1.591	1.236-2.048	<0.0001		2.141	1.743-2.629	<0.0001

PVTT, portal vein tumor thrombus; HGB, haemoglobin; PLT, platelet; ALT, alanine aminotransferase; TBIL, totall bilirubin; AFP, alpha-fetoprotein; Cox proportional hazards model; P < 0.05 was considered as statistically significant.

**Table 4:** Multivariate analysis of risk factors related to overall survival for female and male in BCLC stage 0-B HBV-related HCC patients

	Overall Survival in female					Overall Survival in male				
	Univariate analysis			Multivariate analysis		Univariate analysis			Multivariate analysis	
Variables	HR	95%CI	P values	Hazard Ratio for Death (95%CI)		HR	95%CI	P values	Hazard Ratio for Death (95%CI)	
Age(y)	1.14	0.40-3.23	0.81			0.98	0.72-1.32	0.87		
≤50										
>50										
Smoking	0.63	0.09-4.59	0.65			1.17	0.88-1.55	0.29		
Non-smoker										
Smoker										
Alcohol	2.85	0.68-11.87	0.15			1.27	0.95-1.68	0.1		
No alcohol										
Alcohol										
HBV-DNA(IU/ml)	2.15	1.10-4.20	0.025			1.81	1.36-2.42	<0.0001	1.44(1.07-1.94)	
Low≤500										
High>500										
HBeAg at baseline	1.33	0.67-2.65	0.41			1.01	0.79-1.46	0.64		
Negative										
Positive										
Tumor multiplicity	2.5	1.29-4.84	0.007	2.06(1.05-4.07)		1.87	1.41-2.49	<0.0001	1.48(1.11-1.98)	
Solitary										
Multiple										
Tumor size(cm)	3.97	1.98-7.98	<0.0001	3.37(1.65-6.89)		3.25	2.42-4.35	<0.0001	2.47(1.83-3.34)	
≤5										
>5										
PVTT at baseline	0.049	0.00-647825.1	0.719			3.03	0.97-9.45	0.057		
Yes										
No										
Cirrhosis	0.66	0.20-2.14	0.48			1.25	0.74-2.12	0.41		
Yes										
No										
WBC (10^9^/L)	1.75	0.90-3.39	0.098			1.002	0.75-1.35	0.99		
Low≤4										
High>4										
HGB(g/L)	0.64	0.33-1.26	0.2			0.502	0.37-0.67	<0.0001	0.61(0.45-0.82)	
Low≤120										
High>120										
PLT (10^9^/L)	1.4	0.72-2.73	0.33			1.14	0.85-1.51	0.39		
Low≤100										
High>100										
ALT (U/L)	1.63	0.76-3.47	0.21			1.52	1.13-2.04	0.006		
Normal ≤50										
High>50										
TBIL(μmol/L)	1.65	0.85-3.20	0.14			1.64	1.24-2.19	0.001	1.4(1.05-1.88)	
Normal≤18.8										
High>18.8										
ALB(g/L)	0.46	0.19-1.12	0.09			0.58	0.42-0.80	0.001		
Normal≤40										
High>40										
PTA(%)	1.01	0.47-2.15	0.99			0.59	0.44-0.79	0.001		
Low≤70										
High>70										
AFP(ng/ml)	1.08	0.49-2.38	0.85			3.06	2.28-4.10	<0.0001	2.63(1.95-3.54)	
Low≤400										
High>400										

PVTT, portal vein tumor thrombus; WBC, white blood cell; HGB, haemoglobin; PLT, platelet; AFP, alpha-fetoprotein; ALT, alanine aminotransferase; TBIL, totall bilirubin; ALB, albumin; PTA, prothrombin time activity; Cox proportional hazards model; P < 0.05 was considered as statistically significant.
