# Supplementary material for: Impact of gender as a prognostic factor in HBV-related Hepatocellular Carcinoma: the survival strength of female patients in BCLC stage 0-B
Source: J Cancer. 2019 Jul 10;10(18):4237–44. doi: 10.7150/jca.33430 (PMC6691698; doi:10.7150/jca.33430)
Supplement: Supplementary file 1 — Supplementary figure and tables. [file jcav10p4237s1.pdf]

Table S1.Demographics and characteristics of female and male in BCLC stage 0-B HBV-related HCC patients

Demographic and clinical values	Female n=260(%)	Male n=942(%)	p Value
Age(y)			<0.0001
≤50	33（12.7）	304（32.3）	
＞50	227（87.3）	638（67.7）	
Smoking			<0.0001
Non-smoker	249（95.8）	492（52.2）	
Smoker	11（4.2）	450（47.8）	
Alcohol			<0.0001
No alcohol	254（97.7）	542（57.5）	
Alcohol	6（2.3）	400（42.5）	
HBV-DNA(IU/ml)			0.079
Low≤500	158（60.8）	515（54.7）	
High＞500	102（39.2）	427（45.3）	
HBeAg at baseline			0.642
Negative	178（68.5）	659（70.0）	
Positive	82（31.5）	283（30.0）	
Tumor multiplicity			0.034
Solitary	180（69.2）	585（62.1）	
Multiple	80（30.8）	357（37.9）	
Tumor size(cm)			0.035
≤5	225（86.5）	762（80.9）	
>5	35（13.5）	180（19.1）	
PVTT at baseline			0.965
Yes	2（0.8）	7（0.7）	
No	258（99.2）	935（99.3）	
Cirrhosis			0.079
Yes	244（93.8）	851（90.3）	
No	16（6.2）	91（9.7）	
WBC (10^9/L)			<0.0001
Low≤4	159（61.2）	343（36.4）	
High＞4	101（38.8）	599（63.6）	
HGB(g/L)			<0.0001

Low≤120	123（47.3）	225（23.9）	
High＞120	137（52.7）	717（76.1）	
PLT (10^9/L)			<0.0001
Low≤100	168（64.6）	443（47.0）	
High＞100	92（35.4）	499（53.0）	
ALT (U/L)			0.001
Normal ≤50	213（81.9）	679（72.1）	
High＞50	47（18.1）	263（27.9）	
TBIL(μmol/L)			0.853
Normal≤18.8	162（62.3）	581（61.7）	
High＞18.8	98（37.7）	361（38.3）	
ALB(g/L)			0.056
Normal≤40	183（70.4）	603（64.0）	
High＞40	77（29.6）	339（36.0）	
r-GGT(U/L)			<0.0001
Normal≤60	195（75.0）	509（54.0）	
High＞60	65（25.0）	433（46.0）	
PTA(%)			0.744
Low≤70	68（26.2）	237（25.2）	
High＞70	192（73.8）	705（74.8）	
AFP(ng/ml)			0.373
Low≤400	203（78.1）	759（80.6）	
High＞400	57（21.9）	183（19.4）	

p Value between female and male groups.

PVTT, portal vein tumor thrombus; WBC, white blood cell; RBC, Red blood cell; HGB, haemoglobin; PLT, platelet; CR, creatinine; AFP, alpha-fetoprotein; ALT, alanine aminotransferase; TBIL, totall bilirubin; ALB, albumin; γ-GGT, γ-glutamyl transferase; PTA, prothrombin time activity .

Table S2. Multivariate analysis of risk factors related to overall survival and progression-free survival in our prospective cohort study for BCLC stage 0-B HBV-related HCC patients

Variable	Overall Survival			Progression-free Survival		
	HR	95%CI	P	HR	95%CI	P
Gender						
Male	1			1		
Female	0.336	0.131-0.859	0.023	0.536	0.292-0.985	0.044
Child-Pugh						
A	1			1		
B	1.762	0.965-3.216	0.065	1.782	1.183-2.683	0.006
HBV-DNA(IU/ml)						
Low≤500	1			----		
High＞500	2.923	1.701-5.021	<0.0001	----	----	----
r-GGT(U/L)						
Low≤60	----			1		
High＞60	----	----	----	1.648	1.112-2.442	0.013
HGB(g/L)						
Low≤120	1			----		
High＞120	0.479	0.263-0.874	0.016	----	----	----
AFP(ng/ml)						
Low≤400	1			1		
High＞400	2.155	1.255-3.702	0.005	1.629	1.079-2.460	0.020

γ-GGT, γ-glutamyl transferase; HGB, haemoglobin; AFP, alpha-fetoprotein; Cox proportional hazards model; $P < 0.05$ was considered as statistically significant.

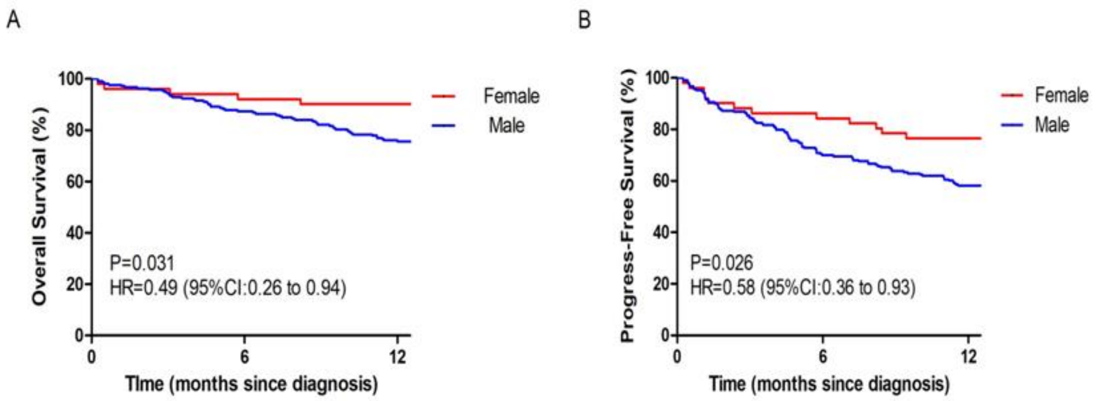

Fig.S1. Overall survival (OS) and Progression-free survival (PFS) in prospective cohort study for BCLC stage 0-B HBV-related HCC patients. (A-B) The OS (A) and PFS (B) in BCLC stage 0-B.
